# Validity of a minimally invasive autopsy tool for cause of death determination in pediatric deaths in Mozambique: An observational study

**DOI:** 10.1371/journal.pmed.1002317

**Published:** 2017-06-20

**Authors:** Quique Bassat, Paola Castillo, Miguel J. Martínez, Dercio Jordao, Lucilia Lovane, Juan Carlos Hurtado, Tacilta Nhampossa, Paula Santos Ritchie, Sónia Bandeira, Calvino Sambo, Valeria Chicamba, Mamudo R. Ismail, Carla Carrilho, Cesaltina Lorenzoni, Fabiola Fernandes, Pau Cisteró, Alfredo Mayor, Anelsio Cossa, Inacio Mandomando, Mireia Navarro, Isaac Casas, Jordi Vila, Khátia Munguambe, Maria Maixenchs, Ariadna Sanz, Llorenç Quintó, Eusebio Macete, Pedro Alonso, Clara Menéndez, Jaume Ordi

**Affiliations:** 1 ISGlobal, Barcelona Centre for International Health Research (CRESIB), Hospital Clinic of Barcelona, Universitat de Barcelona, Barcelona, Spain; 2 Centro de Investigação em Saúde de Manhiça, Maputo, Mozambique; 3 Catalan Institution for Research and Advanced Studies (ICREA), Barcelona, Spain; 4 Department of Pathology, Hospital Clinic of Barcelona, Universitat de Barcelona, Barcelona, Spain; 5 Department of Microbiology, Hospital Clinic of Barcelona, Universitat de Barcelona, Barcelona, Spain; 6 Department of Pathology, Maputo Central Hospital, Maputo, Mozambique; 7 Department of Pediatrics, Maputo Central Hospital, Maputo, Mozambique; 8 Faculty of Medicine, Eduardo Mondlane University, Maputo, Mozambique; 9 Consorcio de Investigación Biomédica en Red de Epidemiología y Salud Pública, Madrid, Spain; Umeå Centre for Global Health Research, Umeå University, SWEDEN

## Abstract

**Background:**

In recent decades, the world has witnessed unprecedented progress in child survival. However, our knowledge of what is killing nearly 6 million children annually in low- and middle-income countries remains poor, partly because of the inadequacy and reduced precision of the methods currently utilized in these settings to investigate causes of death (CoDs). The study objective was to validate the use of a minimally invasive autopsy (MIA) approach as an adequate and more acceptable substitute for the complete diagnostic autopsy (CDA) for pediatric CoD investigation in a poor setting.

**Methods and findings:**

In this observational study, the validity of the MIA approach in determining the CoD was assessed in 54 post-neonatal pediatric deaths (age range: ≥1 mo to 15 y) in a referral hospital of Mozambique by comparing the results of the MIA with those of the CDA. Concordance in the category of disease obtained by the two methods was evaluated by the Kappa statistic, and the sensitivity, specificity, and positive and negative predictive values of the MIA diagnoses were calculated.

A CoD was identified in all cases in the CDA and in 52/54 (96%) of the cases in the MIA, with infections and malignant tumors accounting for the majority of diagnoses. The MIA categorization of disease showed a substantial concordance with the CDA categorization (Kappa = 0.70, 95% CI 0.49–0.92), and sensitivity, specificity, and overall accuracy were high. The ICD-10 diagnoses were coincident in up to 75% (36/48) of the cases. The MIA allowed the identification of the specific pathogen deemed responsible for the death in two-thirds (21/32; 66%) of all deaths of infectious origin. Discrepancies between the MIA and the CDA in individual diagnoses could be minimized with the addition of some basic clinical information such as those ascertainable through a verbal autopsy or clinical record. The main limitation of the analysis is that both the MIA and the CDA include some degree of expert subjective interpretation.

**Conclusions:**

The MIA showed substantial concordance with CDA for CoD identification in this series of pediatric deaths in Mozambique. This minimally invasive approach, simpler and more readily acceptable than the more invasive CDA, could provide robust data for CoD surveillance, especially in resource-limited settings, which could be helpful for guiding child survival strategies in the future.

## Abstract 2

Quique Bassat and colleagues examine the validity of a standardized minimally invasive autopsy approach by comparison with that of the complete diagnostic autopsy in a series of children who died at Maputo Central Hospital in Mozambique.

## Author summary

**Why was this study done?:**

- Child mortality has been steadily decreasing globally in the last 25 years, but nearly 6 million child deaths still occur every year, predominantly in low- and middle-income countries.
- In these settings, current knowledge on the specific causes of child death is jeopardized by the scarcity of good-quality mortality data.
- Complete diagnostic autopsies, indisputably considered the reference method for cause of death investigation, are seldom conducted in poor settings, both on account of their limited acceptability and because of a generalized scarcity of the required pathological technical expertise.
- We aimed to validate a simpler and potentially much more acceptable minimally invasive autopsy (MIA) approach, by comparing its performance against the complete diagnostic autopsy for cause of death investigation in pediatric deaths.

**What did the researchers do and find?:**

- In this study, 54 deaths in Mozambican children <15 years of age were independently studied using both the MIA method and the full autopsy.
- A comparison of the results of both methods was conducted, and concordance assessed.
- The MIA approach produced good-quality tissues and bodily fluids for histopathological and microbiological investigations.
- The MIA categorization of disease showed substantial concordance with the complete diagnostic autopsy, and the diagnostic accuracy of the MIA method was also high.
- Coincidence (matching) of the precise ICD-10 diagnosis between the MIA and the full autopsy was moderate, almost perfect, or perfect in 36/48 of the cases (75%) in which both methods were concordant for general categorization of disease.
- The MIA approach was particularly accurate in the diagnosis of infectious and oncological causes of death, but failed to identify congenital conditions.
- Additionally, the specific microorganisms associated with mortality were readily identified in the majority of MIA-obtained samples.

**What do these findings mean?:**

- The MIA can identify with significant precision and accuracy the ultimate cause of a child’s death and even other underlying conditions.
- Due to its less invasive nature, such a method could be more easily utilized in resource-constrained settings for cause of death investigation and mortality surveillance.
- Reliable estimates of the causes of child mortality can help policy makers design and implement better and more evidence-based preventive strategies to improve child survival in high-burden low-income settings.

## Introduction

In recent decades, the world has witnessed remarkable and unprecedented progress in terms of child survival, with child mortality rates halving in the last 25 years, and the annual number of child deaths dropping from the approximately 12.7 million annual deaths estimated to have occurred in 1990 to less than 5.9 million in the year 2015 [1]. In spite of these encouraging trends, the global health community still faces significant challenges in establishing the causes of these child deaths, particularly for those occurring in low- and middle-income countries, and is eager to find solutions for the significant uncertainty surrounding estimates and attributable causes of mortality [2]. Indeed, the construction of models for cause of death (CoD) estimation has in the past relied, particularly for deaths occurring in poor countries, on data obtained through death certificates, clinical records, and verbal autopsies, all of which have shown important weaknesses, including low coverage, poor specificity, and a high possibility of misclassification errors. This partly explains the major discrepancies found in the different available estimates of the contribution of different diseases and infections to child mortality [3,4]. Lack of clarity in this respect is not helping policy makers, who strongly rely on such estimates for local, national, and global health planning and prioritization [5].

High expectations have recently been placed on the development and validation of a minimally invasive autopsy (MIA) tool [6], an approach based on postmortem sampling of key organs using needle biopsies. This procedure provides the possibility of a thorough histopathological and microbiological investigation of the obtained tissues and fluids and, therefore, more robust and plausible explanations for the CoD than currently utilized non-postmortem methods. The MIA method was designed as a proxy and eventually potential substitute for the complete diagnostic autopsy (CDA). The CDA is considered the gold standard method for CoD ascertainment, but it requires high levels of expertise and infrastructure that are seldom available in low- and middle-income countries and has little or virtually no acceptability in these settings. The MIA, which can be conducted relatively rapidly and barely leaves visible marks, has the potential to be much more acceptable [7], and could be implemented as a surveillance mechanism much more easily than CDA in settings where robust CoD data are scarce but more needed [8].

The MIA tool was recently validated in a series of studies in Mozambique for adult [9], perinatal [10], and maternal deaths. In this validation study, the concordance between the two methods (MIA and CDA) was high (75.9%), and the MIA reliably recognized infectious diseases and cancers as CoD. Here, we aimed to validate the MIA approach against the CDA in a post-neonatal pediatric series of deaths.

## Methods

This observational study received the approval of the Clinical Research Ethics Committee of the Hospital Clinic of Barcelona (Spain; File 2013/8677) and the National Bioethics Committee of Mozambique (Mozambique; Ref. 342/CNBS/13). MIA and CDA procedures were conducted only after verbal informed consent was provided by the relatives.

### Study setting and design

The study was conducted at the Department of Pathology of the Maputo Central Hospital, a 1-500-bed, government-funded quaternary health care center, in collaboration with the Department of Pediatrics, which admits children until the age of 15 y.

The STROBE statement and the prospective analysis plan are included as S1 and S2 Text, respectively. Additional data are available upon request, in accordance with the consortium agreement signed by the CaDMIA project partnership. Data use and transfer are monitored by ISGlobal’s Biostatistics and Data Management Unit (contact email: ubioesdm@isglobal.org).

Child deaths that occurred within the hospital from 7 May 2014 to 9 March 2015 were eligible for inclusion if they fulfilled the following criteria: (1) a CDA requested by the clinician as part of the medical evaluation of the patient and (2) verbal informed consent to perform the autopsy given by the relatives. Neonatal deaths (within first 28 d of life) and deaths of traumatic origin were excluded. A member of the study staff was tasked with liaising with the families in cases of deaths occurring in the pediatric department, but only after the clinicians had asked for consent for postmortem examination.

### Autopsy procedures

Detailed MIA pathological and microbiological methods have been reported elsewhere [11,12]. The procedure, adapted for pediatric deaths, included an initial disinfection of the surface of the body, followed by the collection of blood and cerebrospinal fluid (CSF), using pediatric needles and aiming to collect ~10–15 ml of each fluid. In young infants, a lumbar puncture was attempted first in order to obtain CSF. If unsuccessful, CSF was obtained by occipital puncture, reaching the cisterna magna, as in adults. The MIA procedure also included the puncture of solid organs (liver, lungs, and central nervous system [CNS]) (using biopsy needles, 14G–16G) and bone marrow (using a trephine needle) for microbiological and pathological analysis. In addition, heart, spleen, and kidneys were sampled (using biopsy needles, 14G–16G) for pathology examination. Immediately after the MIA, the CDA procedure was conducted by a second pathologist not involved with the MIA and following a standardized protocol for pediatric autopsies [13]. Histological and microbiological analyses were conducted on samples from the same viscera collected in the MIA and from any grossly identified lesions. The microbiological results for the blood and CSF were also included in the CDA evaluation.

### Histological and microbiological analyses

A team of two pathologists and two microbiologists reviewed and analyzed the samples from the MIA, blind to any clinical information and before the analysis of the CDA samples and revision of the autopsy’s macroscopic findings report, which provides information about the findings during the autopsy process, at both the body and organ level (e.g., malformations, internal hemorrhages, tumors). The histological evaluation included staining with hematoxylin and eosin in all samples and additional histochemical and/or immunohistochemical stains (e.g., Ziehl—Neelsen and cytomegalovirus) whenever needed to reach a diagnosis. Microbiological methods have been reported in detail elsewhere [12]. In all recruited cases, investigation for highly incident pediatric pathogens was conducted. This included screening for *Plasmodium falciparum* by real-time PCR [14,15]; detection of antibodies against human immunodeficiency virus (HIV)–1/2 and against hepatitis C virus; detection of hepatitis B surface antigen; multiplexed PCR analyses for most common respiratory viruses (e.g., respiratory syncytial virus and adenovirus) and bacteria (e.g., *Streptococcus pneumoniae*, *Haemophilus influenzae*, *Neisseria meningitidis*); culture of organisms (bacterial and fungal) using samples from blood, CSF, liver, lungs, and CNS; and, in some cases, further investigation of bacterial or fungal presence using 16S rRNA gene PCR [16] or 18S rDNA—ITS PCR, respectively. All the sequencing reactions for amplicons obtained by either 16S rRNA or 18S rDNA—ITS PCR were performed by the Sanger method at the sequencing platform of the Hospital Clinic of Barcelona. Identification was performed by comparing the sequences obtained with those present in GenBank, using the BLAST algorithm (http://blast.ncbi.nlm.nih.gov/Blast.cgi). HIV viral load was determined in samples positive for antibodies against HIV-1/2, and for patients confirmed to be HIV-infected, an additional microbiological screening was conducted that included real-time PCR in CSF and CNS samples for *Toxoplasma gondii*, *Mycobacterium tuberculosis*, and *Cryptococcus* spp. and real-time PCR in lung samples for *Pneumocystis jirovecii*, *Cryptococcus* spp., and *M*. *tuberculosis*. Other microorganisms were further investigated depending on the pathological findings observed in the MIA-obtained tissues.

After a washout period (minimum 3 mo, range 3–6 mo), the same team analyzed the samples of the CDA following the same approach used for the analysis of the MIA samples, with the only exception that tissues obtained during CDA were not routinely cultured, and only molecular methods were used to investigate pathogens.

As previously described elsewhere [9], two scales were developed to grade the strength of the evidence of the findings, one based on the severity of the pathological findings and the other on the distribution and type of the microorganisms identified (Table 1).

**Table 1:** Strength of the evidence of the autopsy findings in the complete diagnostic autopsy and the minimally invasive autopsy.

Level	Evidence	Pathological findings^a^	Microbiological findings^b^
0	None	No pathological findings, or nonspecific changes	No microorganisms identified
1	Slight	Mild pathological findings, unlikely to be the cause of death	Microorganisms that are frequent contaminants
2	Fair	Mild pathological findings, possibly causing death^c^	Microorganisms that can represent true pathogens or colonization/contaminants; mixed infections^d^
3	Moderate	Pathological findings of moderate intensity, probably causing death^c^	Microorganisms that can represent either true pathogens or colonization/contaminants detected by both molecular and culture-based methods
4	Strong	Severe pathological findings likely to be the cause of death	Microorganisms that represent true pathogens and/or microorganisms consistently detected in ≥4 samples

^a^Pathological findings include only microscopic changes in the minimally invasive autopsy and both macro- and microscopic changes in the complete diagnostic autopsy.

^b^Microbiology examples according to strength of evidence classification: (1) coagulase-negative staphylococci, group viridans streptococci; (2 and 3) Enterobacteriaceae bacteria such as *Klebsiella pneumoniae* or *Escherichia coli*, non-fermentative gram-negative bacilli, adenovirus, parainfluenza virus; (4) *S*. *pneumoniae*, *H*. *influenzae*, *Cryptococcus* spp., *T*. *gondii*, *M*. *tuberculosis*, *P*. *jirovecii*.

^c^The finding in the histological (histochemistry or immunohistochemistry) exam of a microorganism associated with inflammatory changes increased the pathological score by one.

^d^Mixed infection: multiple pathogens are detected, and it is not possible to determine which one represents the etiological cause of death.

Using a combination of the strength of the evidence of the pathological and the microbiological findings, a category was assigned to the certainty of the CoD attribution of the MIA diagnosis and the CDA diagnosis. These categories included no diagnosis and diagnosis of low, moderate, high, and very high certainty (Table 2). In the CDA evaluation, the clinical data were used to provide guidance and/or evidence on CoD in cases with no diagnosis or with pathological/microbiological diagnoses of low or moderate certainty.

**Table 2:** Level of certainty of the diagnosis of cause of death obtained by combination of the strength of the evidence of the pathological and microbiological findings.

Pathology	Microbiology								
	0	1		2		3		4	
		N	Y	N	Y	N	Y	N	Y
0*	No diagnosis*	No diagnosis*		No diagnosis*		Low*		Moderate*	
**1**	Low	Low	Low	Low	Low	Moderate	Moderate	Moderate	Moderate
**2**	Low	Low	Low	Low	Moderate	Moderate	Moderate	Moderate	High
**3**	Moderate	Moderate	Moderate	Moderate	High	High	High	High	Very High
**4**	High	High	High	High	High	High	Very High	Very High	Very High

N: the microorganisms identified are rarely associated with the histological lesions observed; Y: the microorganisms identified are in concordance with the histological lesions observed.

*When the level of evidence for the pathology findings is zero, N and Y are not applicable.

### Determination of the cause of death

Once all the analyses of the MIA samples had been completed, a panel composed of a pathologist, a microbiologist, and a pediatrician with expertise in infectious diseases and epidemiology evaluated all the data of the MIA and assigned the putative MIA diagnosis. Details of the methodology for CoD determination have been described elsewhere [9]. Briefly, the findings obtained in the MIA sampling were used to assign the MIA diagnosis, i.e., the main condition putatively leading to death. No clinical information was used for the MIA diagnosis assignment. Whenever detected, a chain of conditions (up to four) was established for the most probable chronological sequence of events leading to death [17]. Fundamental diseases potentially contributing to the death were classified as underlying conditions (e.g., HIV infection or malnutrition). In all cases, the direct CoD and not the underlying disease was considered as the main CoD (e.g., bacterial pneumonia in a toddler with malnutrition, or lymphoma in an HIV-infected child). Finally, other conditions or concomitant infections contributing to death but not related to the chain of events leading to death were considered as other significant conditions.

The same team evaluated the data from the CDA and assigned the final CDA diagnosis of CoD using the same methodology. The CDA diagnosis integrated all the findings from the macroscopic, histological, and microbiological analysis together with the clinical information and was considered the “gold standard” diagnosis.

All morbid conditions directly leading to death, any underlying conditions (if present), as well as any other significant conditions possibly contributing to death were codified following the International Classification of Diseases, Tenth Revision (ICD-10) [18]. This codification process was conducted independently for the MIA and the CDA diagnoses. The CoDs were classified into five major categories of diseases: (1) infectious diseases, (2) malignant tumors, (3) congenital malformations, (4) other diseases (including noninfectious cardiovascular or pulmonary diseases), and (5) nonconclusive.

### Statistical methods

In the original protocol, a sample size calculation was performed according to the expected prevalence of infectious diseases causing death in all age groups. A minimum sample size was not calculated to assess the concordance between MIA and CDA for all categories of disease in this particular age group. Instead, we used cases available that met our study sample criteria.

The concordance between the MIA diagnosis and the gold standard CDA diagnosis in terms of major category of disease was assessed by the Kappa statistic and was interpreted as suggested by Landis and Koch [19]. The diagnostic accuracy of the MIA, i.e., its ability to identify the categories established by the gold standard CDA diagnosis, was evaluated via sensitivity, specificity, positive and negative predictive values, and total percentage of cases correctly classified.

Additionally, the coincidence of ICD-10 codes between the MIA and the CDA was assessed for each individual case to determine, in those cases where disease categorization was concordant between both methods, to what degree the MIA’s attributed CoD diagnosis coincided with that of the gold standard. The ICD-10 system classifies diagnoses into nested classes, where diseases and conditions are organized in chapters, blocks, and four-character categories [17,18]. Thus, coincidence was classified as perfect when the ICD-10 codes were identical in chapter, block, and four-character category between the MIA and CDA diagnosis [17], and “almost perfect” when ICD-10 codes coincided up to the first three characters of the category. Coincidence was classified as moderate when the codes were within the same chapter and block, but there was a discrepancy in the first three characters of the category. Coincidence was classified as low when the codes were within the same chapter, but there was a discrepancy in the block and in the first three characters of the category. Finally, when the MIA and the CDA diagnoses were in a different chapter, the coincidence in diagnosis was classified as “none.”

Statistical analyses were performed using Stata version 14.1 (StataCorp, College Station, Texas, US). The analytical plan was determined once all the histological and microbiological results became available.

## Results

**Fig 1:**
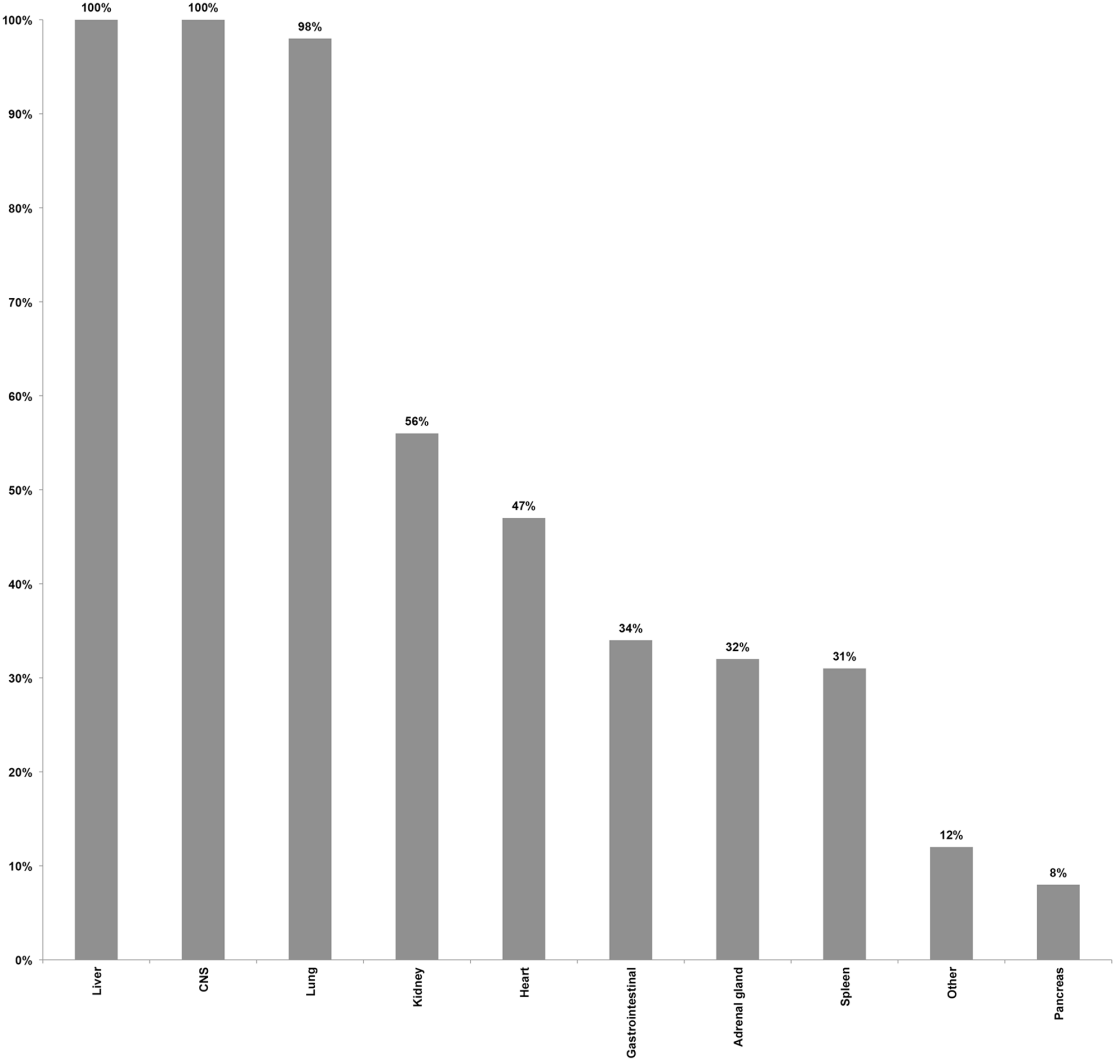
Performance of the minimally invasive autopsy method in terms of successful sampling of each targeted tissue/organ in the 54 studied pediatric cases. CNS, central nervous system.

Coupled MIA and CDA procedures were performed in 54 children (37 males [69%] and 17 females [32%]). Mean age was 5.6 y (range: ≥1 mo–15 y), with 14/54 (26%) being infants, and 17/54 (32%) being between 12 and 59 mo of age. The interval between death and the beginning of postmortem procedures ranged between 4 and 56 h, with 19/54 (35%) procedures being conducted after 24 h, of which 2/54 (4%) were performed over 48 h after the time of death. All CDAs were conducted within 30 min after completing the MIA. Blood was successfully obtained in 54/54 (100%) of the cases, with a mean volume of 18.3 ml (standard deviation 3.1 ml), and CSF was obtained in 53/54 (98%) of the cases. Liver and CNS were successfully obtained by MIA puncture in 100% of the cases, and lung in 53/54 (98%) of the cases. Lower rates of success were obtained for kidney, heart, and spleen. In addition to the targeted organs, gastrointestinal tract, adrenal glands, pancreas, and other organs, although not deliberately targeted, were also often sampled as part of the routine thoracic and abdominal punctures. Fig 1 summarizes the performance of the MIA method in terms of successful sampling of each targeted tissue/organ in the 54 studied cases.

### Minimally invasive autopsy and complete diagnostic autopsy diagnoses

A final CoD was identified in the CDA in 100% (54/54) of the cases: 44/54 (82%) with high or very high certainty, 7/54 (13%) with moderate certainty, and 3/54 (6%) with low certainty. Infections accounted for 78% of the deaths (42/54), malignant tumors for 13% (7/54), congenital malformations for 4% (2/54), and other diseases for 6% (3/54, including one case each of heart failure, acute interstitial pneumonia, and cerebral hemorrhage). Within the category of infectious diseases, disseminated infections (including sepsis of different etiologies and miliary tuberculosis), accounted for 16/42 cases, followed by pulmonary infections (13/42) and CNS infections (8/42). Within the category of malignant tumors, three of the seven cases were Burkitt lymphoma and two were Ewing sarcoma/primitive neuroectodermal tumors.

A CoD was identified in the MIA in 52/54 (96%) of the cases: 37/52 (71%) with high or very high certainty, 11/52 (21%) with moderate certainty, and 4/52 (8%) with low certainty. Infections accounted for 42/54 (78%) of the deaths, malignant tumors for 7/54 (13%), other diseases for 3/54 (6%), and in two cases (4%) the MIA diagnosis was nonconclusive. S1 Table summarizes the CoD for each individual according to the MIA and the CDA.

### Concordance in the categorization of disease between the minimally invasive autopsy diagnosis and the complete diagnostic autopsy diagnosis

Table 3 shows the concordance of the categorization of the CoD established by the MIA and the CDA (gold standard). The MIA categorization of disease showed substantial concordance with the CDA categorization (Kappa = 0.70, 95% CI 0.49–0.92) and agreed in 48/54 (89%) of the cases. Concordance in the infectious disease (39/42; 93%) and malignant tumor (7/7; 100%) categories was high, while the MIA did not agree with the CDA in the two cases of congenital malformations (0/2) and agreed in only one of the three cases classified in the “other diseases” category (33%).

**Table 3:** Concordance of the categorization of the cause of death established by the minimally invasive autopsy and the complete diagnostic autopsy (gold standard).

MIA diagnosis	CDA diagnosis (gold standard)					
	Infectious diseases	Malignant tumors	Congenital malformations	Other diseases	Nonconclusive	Total
Infectious diseases	**39**	0	2	1	0	42 (78%)
Malignant tumors	0	**7**	0	0	0	7 (13%)
Congenital malformations	0	0	**0**	0	0	0 (0%)
Other diseases	1	0	0	**2**	0	3 (6%)
Nonconclusive	2	0	0	0	**0**	2 (4%)
Total	42 (78%)	7 (13%)	2 (4%)	3 (6%)	0 (0%)	**54 (100%)**

Kappa: 0.70 (standard error 0.0956; 95% CI 0.49–0.92), which is considered substantial concordance. The numbers in bold represent the concordant cases between both methods.

CDA, complete diagnostic autopsy; MIA, minimally invasive autopsy.

### Coincidence of ICD-10 codes between the minimally invasive autopsy and the complete diagnostic autopsy for identification of the cause of death

For the 48 cases in which no discrepancy was found in categorization of disease between the MIA and the CDA, we performed an additional analysis to evaluate the degree of coincidence of ICD-10 codes (from perfect to none) between the two methods. Table 4 summarizes the coincidence spectrum regarding ICD-10 coding, according to disease category. The coincidence level was generally good, with 36 of the 48 cases (75%) showing a perfect, almost perfect, or moderate coincidence.

**Table 4:** Summary of ICD-10 code coincidence for the 48 cases for which disease categorization did not show discrepancy between the minimally invasive autopsy and the complete diagnostic autopsy diagnosis.

Disease category (concordant cases)	Coincidence in diagnosis (MIA versus CDA)							
	Perfect or almost perfect		Moderate		Low		None	
	*n*	Percent (95% CI)	*n*	Percent (95% CI)	*n*	Percent (95% CI)	*n*	Percent (95% CI)
Overall (*n* = 48)	32	66.7 (45.0–72.4)	4	8.3 (2.1–17.9)	3	6.3 (1.2–15.4)	9*	18.8 (7.9–29.3)
Infectious diseases (*n* = 39)	24	61.5 (30.9–58.6)	3	7.7 (1.2–15.4)	3	7.7 (1.2–15.4)	9	23.1 (7.9–29.3)
Malignant tumors (*n* = 7)	7	100.0 (5.4–24.9)	0	0 (0–6.6)^†^	0	0 (0–6.6)^†^	0	0 (0–6.6)^†^
Other diseases (*n* = 2)	1	50.0 (0.1–9.9)	1	50.0 (0.1–9.9)	0	0 (0–6.6)^†^	0	0 (0–6.6)^†^

*The nine cases in which ICD-10 code coincidence was categorized as “none” involved the following pairs of discordant ICD-10 chapter ascertainments: (1) CDA: other bacterial diseases; MIA: influenza and pneumonia; (2) CDA: other bacterial diseases; MIA: influenza and pneumonia; (3) CDA: other bacterial diseases; MIA: influenza and pneumonia; (4) CDA: viral infections of the central nervous system; MIA: influenza and pneumonia; (5) CDA: influenza and pneumonia; MIA: other bacterial diseases; (6) CDA: diseases of peritoneum; MIA: influenza and pneumonia; (7) CDA: diseases of peritoneum; MIA: influenza and pneumonia; (8) CDA: diseases of peritoneum; MIA: other bacterial diseases; (9) CDA: renal tubule-interstitial diseases; MIA: influenza and pneumonia.

^†^One-sided 97.5% confidence interval.

CDA, complete diagnostic autopsy; MIA, minimally invasive autopsy.

In children dying from infectious diseases, the CDA detected a plausible microorganism to cause the infection in 32/42 (76%) cases. The MIA detected and assigned the same etiological agent in 21/32 (66%) of these cases. Table 5 shows the list of etiological agents identified in the CDA and MIA, and in which number of cases the same etiological agent was identified for the same CoD diagnosis. As an example, disseminated infection due to gram-negative bacteria was diagnosed in five CDA cases and in five MIA cases, but only in three cases did the diagnoses match for both autopsy methods.

**Table 5:** List of etiological agents identified in the complete diagnostic autopsy and the number of the same identical agents identified in the minimally invasive autopsy, classified by disease category.

Disease category and etiological agent	CDA (gold standard)	MIA	MIA and CDA
**Disseminated infections**	**17**	**17**	**8**
Gram-negative bacteria	5*	5^†^	3
*Streptococcus pneumoniae*	3	3	2
*Mycobacterium tuberculosis*	3	1	1
Cytomegalovirus	2	2	1
*Plasmodium falciparum*	2	1	1
*Haemophilus influenzae*	1	0	0
Tetanus^‡^	1	0	0
*Salmonella enterica*	0	2	0
*Tropheryma whipplei*	0	1	0
No agent	0	2	0
**Pulmonary infections**	**13**	**20**	**11**
Cytomegalovirus	3	3	3
*Streptococcus pneumoniae*	3	6	3
*Pneumocystis jirovecii*	2	2	2
Adenovirus	1	1	1
*Klebsiella pneumoniae*	1	2	0
*Escherichia coli*	0	1	0
*Haemophilus influenzae*	0	1	0
*Mycobacterium tuberculosis*	0	1	0
No agent	3	3	2
**Central nervous system infections**	**8**	**5**	**3**
*Streptococcus pneumoniae*	2	3	2
Rabies virus	2	0	0
*Cryptococcus* sp.	1	1	1
*Acinetobacter baumannii*	0	1	0
No agent	3	0	0
**Other infections**	**4**	**0**	**0**
*Klebsiella pneumoniae*^§^	1	0	0
No agent^‖^	3	0	0
**Total**	**42**	**42**	**22**

*Two cases of *Pseudomonas aeruginosa*, two cases of *E*. *coli*, and one case of *Aeromonas jandaei*.

^†^One case of *A*. *jandaei*, one case of *Ps*. *aeruginosa*, one case of *E*. *coli*, one case of *K*. *pneumoniae*, and one case of mixed Gram-negative infection.

^‡^Tetanus diagnosed by the combination of clinical information and histology findings without microbiological confirmation of the microorganism.

^§^One case of peritonitis.

^‖^Two cases of peritonitis and one case of pyelonephritis.

CDA, complete diagnostic autopsy; MIA, minimally invasive autopsy.

### Accuracy of the minimally invasive autopsy

Table 6 shows the sensitivity, specificity, and the positive and negative predictive values of the MIA diagnosis for the major CoD categories, as well as the percentage of cases correctly classified (accuracy) by the MIA. Sensitivity of the MIA method was high for infectious diseases and malignant tumors, and low or very low for the other categories. Overall accuracy remained high (>89%) for all disease categories.

**Table 6:** Sensitivity, specificity, positive and negative predictive values, and accuracy of the minimally invasive autopsy for the different cause of death categories in children.

Cause of death category	Number of Cases	Sensitivity	Specificity	Positive predictive value	Negative predictive value	Correctly classified
Infectious diseases	42	93 (81, 99)	75 (43, 95)	93 (81, 99)	75 (43, 95)	89 (77, 96)
Malignant tumors	7	100 (59,100)	100 (92,100)	100 (59,100)	100 (92,100)	100 (93,100)
Congenital malformations	2	0 (0,84)	100 (93,100)	N/A	96 (87,100)	96 (87,100)
Other diseases	3	67 (9,99)	98 (90,100)	67 (9,99)	98 (90,100)	96 (87,100)
Nonconclusive	0	N/A	96 (87,100)	0 (0,84)	100 (93,100)	96 (87,100)

Data given as percentage (95% confidence interval).

N/A, not applicable.

### Underlying conditions and concomitant infections

Antibodies against HIV were detected in 17/54 (32%) of the deaths. HIV-1 RNA was detectable in all 17 cases (100%), with viral loads ranging from 463 up to 10,000,000 copies/ml. HIV was considered in all cases as the underlying, but not the direct, CoD. Five cases (9%) had hepatitis B surface antigen antibodies, and one (2%) had antibodies against hepatitis C. *P*. *falciparum* infections were detected by real-time PCR in 4/54 (7%) of the children, but only two patients were deemed to have died from malaria, according to the pathology findings. Additionally, five patients had histological evidence of past malarial infection (diagnosed by the identification of malarial pigment in the liver without blood parasitemia): two of them had Burkitt lymphoma as CoD. Respiratory viruses such as parainfluenza, influenza, adenovirus, and rhinovirus were identified in 14/54 cases (26%). However, adenovirus was the only virus recognized as the plausible etiological agent of a pneumonia that was considered the CoD.

## Discussion

This study shows, to our knowledge for the first time, the validity of a simplified MIA approach, when compared to the gold standard method (CDA plus clinical information), for CoD investigation in pediatric deaths. This validation study shows a substantial degree of concordance (89%; Kappa value of 0.70) between the MIA and the CDA diagnoses in a series of 54 children who died at the referral quaternary hospital in Maputo, Mozambique.

Although the study was designed exclusively to validate a methodology (the MIA) and not to comprehensively describe the etiologies responsible for child death in such a setting, it also provides a glimpse of CoD distribution, with infectious diseases accounting for over three-quarters of all deaths, of which bacterial infections and immunosuppression-derived complications were the most frequently detected. In this respect, our results highlight the brutal impact of the HIV pandemic, blatantly patent for the past decade in Mozambique, as HIV co-infection was confirmed in nearly 28% of all patients.

Malaria—not expected to be a major contributor to mortality in this urban setting, where transmission intensity is among the lowest in the country [20]—was directly responsible for two deaths (4%) and was found as an associated condition in two additional cases. Importantly, nine (17%) of the studied children were confirmed to have died of invasive bacterial infections (sepsis, meningitis, or pneumonia) secondary to either *S*. *pneumoniae* or *H*. *influenzae* type b, two pathogens for which vaccination with conjugate vaccines was already being implemented during the study period as part of the Mozambican Expanded Programme on Immunization.

When assessing the discrepancies observed between the MIA and the CDA diagnoses, it seems apparent that the MIA performs well in the detection of pediatric infections and cancers, both when these are disseminated and—although to a lesser extent—when they are more localized. Pulmonary infections only affecting a specific lobe may be missed if the sampling of the lungs does not involve multiple punctures from both sides. However, in this series only one of the 13 pulmonary infections (8%) was missed, in comparison to the higher proportion (29%) of episodes missed in the adult MIA validation series [9]. Similarly, CNS infections preferentially affecting the occipital and/or brain stem regions may be missed with the current MIA protocol, which uses a transethmoidal approach [11]. However, although infections such as rabies preferentially localize to the brain stem [21], we were able to retrospectively confirm the presence of the virus in tissues obtained with the MIA in two cases in which the diagnosis was originally missed. In children, the often more rapid dissemination of initially localized infections [22,23], in comparison to adults, may prove a comparative advantage in terms of infection detectability with a method such as the MIA, in addition to the fact that, due to the smaller surface area of children, the MIA obtains proportionately larger samples of the organs.

Importantly, none of the 54 studied child deaths were attributed to diarrheal diseases by the MIA or CDA, even though diarrheal disease still accounts for 9% of all deaths in children under 5 y of age (558,000 per year) [24], and up to a fourth of the patients (14/54) were admitted with this symptom in the clinical episode that led to their death. A more systematic pathogen screening of stools, and targeted bowel mucosa biopsies, could be included in the MIA sampling standard operating procedure in order to improve these results. However, further studies are necessary to evaluate the usefulness of stool analysis in postmortem studies. Other important infections that were also missed by the MIA were those of abdominal origin (peritonitis), which in the absence of specifically targeted sampling may be challenging to unveil. The same proposed additions to investigate diarrheal deaths may contribute to improving the detection of abdominal infections by the MIA.

The MIA also missed two types of lethal infections—rabies (two cases) and tetanus (one case)—that appear challenging to ascertain in the absence of any clinical history. As the study aimed to validate the MIA method without any additional clinical information against the gold standard (CDA, including clinical data), it would have been impossible to ascertain such diseases through the limited tissue samples of the MIA in the absence of clear pathognomonic pathological changes. The CDA procedure, which included a summary of the premortem clinical information, was able to confirm such infections after the suspicion was raised by the clinical history. Once the MIA—CDA comparison had been done, rabies and tetanus were also identified in the MIA samples, proving the value of including these pathogens in the MIA diagnostic screening panel. Our purist approach of validation is needed for understanding the performance of the method per se, but it is likely that in the future, should this methodology be implemented, the clinical history—or, in its absence, data from the verbal autopsy—may contribute to the overall diagnosis, if available.

The MIA performed poorly for the detection of congenital malformations (two cardiac malformations) and other noninfectious conditions. The blind sampling scheme of the MIA protocol does not allow the macroscopic observation that becomes possible as part of the CDA, and complementary methods will need to be devised if conditions affecting cardiac morphology and functionality are to be adequately detected through noninvasive postmortem approaches.

A third of the MIAs conducted in the study failed to start within 24 h after death, as proposed in the protocol. The study team accepted these protocol violations in the understanding that data provided in these cases could also be informative for future implementation of the MIA tool for surveillance purposes. Indeed, in a country such as Mozambique, a paradigmatic example of a low-income setting, over 50% of child deaths occur at home [25] and are not necessarily notified or detected. If the MIA tool has been designed for use not only at the health system level but also at the community level, it will need to demonstrate that performance does not significantly decay beyond the first 24 h after death, assuming that in many cases it may be extremely challenging to reach the deceased children in the first 24 h. An increased detection of bacteria such as those belonging to the family Enterobacteriaceae or the genus *Pseudomonas*, traditionally associated with the usual microbiome in forensic samples and usually reaching a higher percentage of recovery between the third and fifth postmortem days [26], would be foreseen if samples are affected by the time difference between death and MIA performance. In our study, only 13% (7/54) of the CoDs were attributed to an infectious agent belonging to one of these bacterial groups; in six out of seven of those cases, the necropsy had been performed within the first 24 h after death. In any case, more data are needed on the performance decay of MIAs after the first 24 h after death, before widening the time window in which MIA can be recommended, particularly if no refrigeration of the corpse is available.

Although the value of the MIA for CoD investigation in more ill-defined diseases such as malnutrition and anemia still needs to be determined, we have shown the potential of this approach to substitute for the CDA for CoD investigation in adults [9], children, and neonates and stillborn infants [10]. However, the MIA sampling technique does not bypass many of the challenges that CoD investigation faces in low- and middle-income countries, including the need for trained technicians, facilities for performance of the procedure, adequately run histology laboratories, high-quality clinical laboratory support, pathology expertise in histological interpretation, and, finally, interdisciplinary collaboration to determine the most likely CoD. Additionally, the MIA could remain a futile advance unless its acceptability and feasibility is shown to be superior to that of the CDA. We have shown that the willingness to know the CoD and hypothetical acceptability of the use of such a tool is high (>75%) at five sites in sub-Saharan Africa and Asia that vary in their geographic, religious, and socio-cultural features [7]. This is a pivotal finding for the MIA’s future implementation as a tool for CoD surveillance, but further socio-behavioral research and guidance are warranted prior to real-life implementation, particularly in rural settings. The widespread and routine implementation of the MIA for CoD surveillance remains, for the moment, quixotic, but this approach could be progressively deployed in sentinel sites, and as a complement to already existing—albeit imperfect—methods, such as the verbal autopsy [27]. However, a methodology like this one, utilizing such a panoply of different microbiological tests and highly specialized histopathological procedures, is probably unpractical outside of research contexts. Its wider implementation for CoD surveillance purposes in those settings where child mortality remains high, but where diagnostic tools are scarce, will require a future simplification of the procedure, its linkage with cheaper point-of-care screening methodologies, and a significant investment in local pathology and microbiology capacity building.

The scaling up of such simplified postmortem methods could then provide robust and credible information from settings where mortality data are patchy or nonexistent, and in turn help refine the rusty estimation models and methods that are routinely utilized to construct CoD global estimates, and which too often have been questioned. The strengthening of data sources would finally result in a more evidence-based public health policy, necessary for continuing progress in the resolution of the global inequities that hamper child survival.

## Supporting information

S1 Table Complete diagnostic autopsy and minimally invasive autopsy results for each case. The table includes the concordance between the two methods in terms of the disease category and the coincidence of ICD-10 coding.
(DOCX)

S1 Text STROBE checklist for the observational study developed in Mozambique to validate the minimally invasive autopsy for cause of death determination in adults. (DOCX)

S2 Text Prospective analysis plan on pathological and microbiological procedures used during the study. (DOCX)

## Abbreviations

CDA: complete diagnostic autopsy
CNS: central nervous system
CoD: cause of death
CSF: cerebrospinal fluid
ICD-10: International Classification of Diseases, Tenth Revision
MIA: minimally invasive autopsy
