# Supplementary material for: Validity of a minimally invasive autopsy tool for cause of death determination in pediatric deaths in Mozambique: An observational study
Source: PLoS Med. 2017 Jun 20;14(6):e1002317. doi: 10.1371/journal.pmed.1002317 (PMC5478091; doi:10.1371/journal.pmed.1002317)
Supplement: S1 Table — The table includes the concordance between the two methods in terms of the disease category and the coincidence of ICD-10 coding. (DOCX) [file pmed.1002317.s001.docx]

| **Case** | **General characteristics** | | | **Time from death until MIA (h)** | **Causes of death** | | | | |  |  |  |
| --- | --- | --- | --- | --- | --- | --- | --- | --- | --- | --- | --- | --- |
|  | **Age** | **Gender** | **HIV status** |  | **Complete autopsy (Gold standard)** | | | **Minimally invasive autopsy** | | | **Concordant in terms of diagnostic category** | **Coincidence in terms of ICD-10 coding hierarchy** |
|  |  |  |  |  | **Diagnosis** | **ICD10** | **Level of certainty*** | **Diagnosis** | **ICD10** | **Level of certainty** |  |  |
|  |  |  |  |  | **Infectious diseases** | | | | | | | |
| 1 | 10 | M | - | 18.3 | Sepsis (*A. jandaei*) | A41.5 | High (2+4) | Sepsis (*A. jandaei*) | A41.5 | Moderate (1+4) | Yes | Perfect |
| 2 | 10 | M | - | 64.5 | Sepsis (*P. aeruginosa*) | A41.5 | High (3+2) | Sepsis (Non fermentative Gram negative bacteria) | A41.5 | High (4+2) | Yes | Perfect |
| 3 | 1 | M | + | 28.8 | Sepsis (*S. pneumoniae*) | A40.3 | Very high (4+4) | Sepsis (*S. pneumoniae*) | A40.3 | Very high (3+4) | Yes | Perfect |
| 4 | 3 | M | NA | 19.8 | Cerebral malaria | B50.0 | Very high (4+4) | Cerebral malaria | B50.0 | Very high (4+4) | Yes | Perfect |
| 5 | 7 | M | - | 20.8 | Sepsis (*P. aeruginosa*) | A41.5 | High (4+2) | Sepsis (*P. aeruginosa*) | A41.5 | Low (1+2) | Yes | Perfect |
| 6 | 3 m | F | + | 6.5 | Disseminated cytomegalovirus | B25.9 | Very high (4+4) | Disseminated cytomegalovirus | B25.9 | Very high (4+4) | Yes | Perfect |
| 7 | 6 | M | NA | 28.8 | Miliary tuberculosis | A19 | Very high (4+4) | Miliary tuberculosis | A19 | Very high (4+4) | Yes | Perfect |
| 8 | 11m | M | - | 27.3 | Sepsis (*E. coli*) | A41.5 | Low (1+3) | Sepsis (*E. coli*) | A41.5 | Low (1+2) | Yes | Perfect |
| 9 | 5 | F | - | 15.6 | Necrotizing pneumonia (no agent) | J18 | High (4+1) | Pneumonia (no agent) | J18 | High (4+1) | Yes | Perfect |
| 10 | 11 | M | - | 21.8 | Pneumonia (no agent) | J18 | Moderate (2+4) | Pneumonia (no agent) | J18 | Moderate (3+1) | Yes | Perfect |
| 11 | 1 | F | + | 13 | Pneumonia (cytomegalovirus) | B25.0 | Very high (4+4) | Pneumonia (cytomegalovirus) | B25.0 | High (4+0) | Yes | Perfect |
| 12 | 4 | M | - | 55.6 | Pneumonia (*S. pneumoniae*) | J13 | Very high (4+4) | Pneumonia (*S. pneumoniae*) | J13 | Very high (4+4) | Yes | Perfect |
| 13 | 3m | F | + | 11.8 | Pneumonia (*P. jiroveci*) | B59 | Very high (4+4) | Pneumonia (*P. jiroveci*) | B59 | High (3+4) | Yes | Perfect |
| 14 | 5m | F | + | 17 | Pneumonia (cytomegalovirus) | B25.0 | Very high (4+4) | Pneumonia (cytomegalovirus) | B25.0 | Very high (4+4) | Yes | Perfect |
| 15 | 5m | M | + | 7.1 | Pneumonia (*P. jirovecii*) | B59 | Very high (4+4) | Pneumonia (*P. jiroveci*) | B59 | Very high (4+4) | Yes | Perfect |
| 16 | 11m | M | - | 32.5 | Pneumonia (Adenovirus) | J12.0 | Very high (3+4) | Pneumonia (Adenovirus) | J12.0 | Moderate (0+4) | Yes | Perfect |
| 17 | 8m | M | + | 27.5 | Pneumonia (cytomegalovirus) | B25.0 | Very high (4+4) | Pneumonia (cytomegalovirus) | B25.0 | Very high (4+4) | Yes | Perfect |
| 18 | 6 | M | - | 29.8 | Meningoencephalitis (*Cryptococcus sp*.) | B45.1 | High (4+2) | Meningoencephalitis (*Cryptococcus sp*.) | B45.1 | High (4+0) | Yes | Perfect |
| 19 | 1M | M | - | 17.3 | Meningitis (*S. pneumoniae*) | G00.1 | Very high (3+4) | Meningitis (*S. pneumoniae*) | G00.1 | High (2+4) | Yes | Perfect |
| 20 | 7 | M | + | 28.7 | Sepsis (*S. pneumoniae*) | A40.3 | Very high (4+4) | Sepsis (*S. pneumoniae*) | A40.3 | Very high (4+4) | Yes | Perfect |
| 21 | 4 | M | - | 24.5 | Granulomatous meningoencephalitis (no agent) | G04 | High (4+0) | Granulomatous meningoencephalitis (*A. baumannii*) | G04.2 | High (4+1) | Yes | Perfect |
| 22 | 6 | M | - | 28.3 | Meningitis (*S. pneumoniae*) | G00.1 | Very high (4+4) | Meningitis (*S. pneumoniae*) | G00.1 | Very high (4+4) | Yes | Perfect |
| 23 | 5m | M | - | 47 | Miliary tuberculosis | A19 | Very high (4+4) | Pulmonary tuberculosis | A15 | Very high (4+4) | Yes | Moderate |
| 24 | 11 | F | - | 22.3 | Sepsis (*H. influenzae*) | A41.3 | Very high (4+4) | Pneumonia (*H. influenzae*) | J14 | Very high (4+4) | Yes | None |
| 25 | 2 | F | - | 20.7 | Pneumonia (*S. pneumoniae*) | J13 | Very high (4+4) | Pneumonia (*S. pneumoniae*) | J13 | Very high (4+4) | Yes | Perfect |
| 26 | 1 | M | + | 22.0 | Pneumonia (no agent) | J18 | Very high (4+4) | Pneumonia (*E. coli*) | J15.6 | High (4+2) | Yes | Moderate |
| 27 | 6M | M | + | 27.4 | Pneumonia (*S. pneumoniae*) | J13 | Very high (4+4) | Pneumonia (*S. pneumoniae*) | J13 | Very high (4+4) | Yes | Perfect |
| 28 | 9 | M | - | 4 | Encephalitis (no agent) | G04 | Low (2+0) | Meningitis (*S. pneumoniae*) | G00.1 | Very high (4+4) | Yes | Moderate |
| 29 | 4m | M | + | 24.1 | Disseminated cytomegalovirus | B25.9 | Moderate (2+4) | Sepsis (*Salmonella enterica*) | A02.8 | Moderate (0+4) | Yes | Low |
| 30 | 4 | F | - | 11.8 | Miliary tuberculosis | A19 | Very high (4+4) | Sepsis (no agent) | A41.9 | High (4+2) | Yes | Low |
| 31 | 2 | M | + | 11 | Sepsis of renal origin (*E. coli*) | A41.5 | High (4+2) | Disseminated cytomegalovirus | B25.9 | Moderate (0+4) | Yes | Low |
| 32 | 13 | M | - | 46 | Sepsis (*S. pneumoniae*) | A40.3 | Very high (4+4) | Pneumonia (*S. pneumoniae*) | J13 | High (2+4) | Yes | None |
| 33 | 2m | M | - | 22 | Bronchopneumonia (*K. pneumoniae*) | J15.0 | Low (2+2) | Sepsis (no agent) | A41.9 | Low (2+1) | Yes | None |
| 34 | 12 | F | - | 25.7 | Rabies | A82 | High (2+4) | Pneumonia (*S. pneumoniae*) | J14 | Moderate (2+2) | Yes | None |
| 35 | 8 | F | + | 20.7 | Tetanus | A35 | High (4+1) | Pneumonia (*S. pneumoniae*) | J13 | Very high (3+4) | Yes | None |
| 36 | 1 | M | - | 24.7 | Peritonitis (no agent) | K65 | High (4+1) | Pneumonia (no agent) | J18 | High (4+1) | Yes | None |
| 37 | 11 | F | - | 17.4 | Pyelonephritis (no agent) | N10 | High (4+3) | Pneumonia (*K. pneumoniae*) | J15.0 | High (3+2) | Yes | None |
| 38 | 1 | F | - | 16.1 | Peritonitis (*K. pneumoniae*) | K65.0 | High (4+2) | Pneumonia (*K. pneumoniae*) | J15.0 | High (4+2) | Yes | None |
| 39 | 9m | M | + | 18.4 | Peritonitis (no agent) | K65.0 | Moderate (3+2) | Sepsis (*K. pneumoniae*) | A41.5 | Moderate (2+2) | Yes | None |
| 40 | 2 | M | - | 26.3 | Malaria | B50.9 | Moderate (1+3) | Pulmonary hemorrhage | R04.8 | Low (2+1) | No | No |
| 41 | 2 | M | - | 13.3 | Rabies | A82 | Moderate (2+4) | Non-conclusive | R99 | No diagnosis (0+1) | No | No |
| 42 | 6 | F | + | 20.8 | Meningoencephalitis (no agent) | G04 | Moderate (3+1) | Non-conclusive | R99 | No diagnosis (0+1) | No | No |
|  |  |  |  |  | **Malignant tumors** | | | | | | | |
| 43 | 7 | M | + | 21.1 | Burkitt's lymphoma (Epstein-Barr virus) | C83.7 | Very high (4+4) | Burkitt's lymphoma (Epstein-Barr virus) | C83.7 | Very high (4+4) | Yes | Perfect |
| 44 | 10 | M | - | 25.1 | Ewing sarcoma- PNET | C49 | High (4+0) | Ewing sarcoma-PNET | C49 | High (4+0) | Yes | Perfect |
| 45 | 12 | F | - | 15.1 | Astrocytoma | C71.9 | High (4+0)) | Astrocytoma | C71.9 | High (4+0) | Yes | Perfect |
| 46 | 12 | M | - | 15.2 | Burkitt's lymphoma (Epstein-Barr virus) | C83.7 | Very high (4+4) | Burkitt's lymphoma (Epstein-Barr virus) | C83.7 | Very high (4+4) | Yes | Perfect |
| 47 | 15 | M | - | 11.9 | Disseminated Ewing sarcoma- PNET | C49 | High (4+0) | Disseminated Ewing sarcoma- PNET | C49 | High (4+0) | Yes | Perfect |
| 48 | 4 | M | - | 51.2 | Burkitt's lymphoma (Epstein-Barr virus) | C83.7 | Very high (4+4) | Burkitt's lymphoma (Epstein-Barr virus) | C83.7 | Very high (4+4) | Yes | Perfect |
| 49 | 14 | F | + | 14.8 | Lymphoblastic leukemia | C91.0 | High (4+0) | Lymphoblastic leukemia | C91.0 | High (4+0) | Yes | Perfect |
|  |  |  |  |  | **Congenital malformations** | | | | | | | |
| 50 | 1m | M | - | 21.3 | Congenital heart disease (Interventricular canal) | Q21.0 | High (4+2) | Sepsis (*Salmonella enteritidis*) | A02.0 | High (4+2) | No | No |
| 51 | 4 | F | - | 6.9 | Congenital heart disease (tetralogy of Fallot) | Q21.3 | High (4+0) | *T. whipplei* infection | K90.8 | Moderate (0+4) | No | No |
|  |  |  |  |  | **Other diseases** | | | | | | | |
| 52 | 2 | M | - | 13.8 | Cerebral hemorrhage. Hemophilia | D66 | High (4+0) | Sepsis (*S. pneumoniae*) | A40.3 | Moderate (1+4) | No | No |
| 53 | 12 | F | - | 10.7 | Acute interstitial pneumonia | J84 | Moderate (3+1) | Acute interstitial pneumonia | J84 | Moderate (3+0) | Yes | Perfect |
| 54 | 14 | M | - | 26.3 | Dilated cardiomyopathy | I50.9 | Very high (4+4) | Suggestive of cardiovascular disease | I51.6 | Moderate (3+1) | Yes | Moderate |

*The level of certainty, ranging from low to very high, is a composite of the two scales designed to measure the strength of the evidence: First number is related to the severity of the pathological findings; second number is related to the distribution and type of the microorganisms identified.
